# Are Astrocytes the Predominant Cell Type for Activation of Nrf2 in Aging and Neurodegeneration?

**DOI:** 10.3390/antiox6030065

**Published:** 2017-08-18

**Authors:** Jeffrey R. Liddell

**Affiliations:** Department of Pathology, The University of Melbourne, Parkville, VIC 3010, Australia; jliddell@unimelb.edu.au; Tel.: +61-3-9035-6041; Fax: +61-3-9349-4004

**Keywords:** astrocyte, neuron, microglia, oligodendrocyte, Keap1, mouse, electrophile, dimethyl fumarate, Cu^II^(atsm), sulforaphane

## Abstract

Nuclear factor erythroid 2-related factor 2 (Nrf2) is a transcription factor that regulates hundreds of antioxidant genes, and is activated in response to oxidative stress. Given that many neurodegenerative diseases including Alzheimer’s disease, Parkinson’s disease, amyotrophic lateral sclerosis, Huntington’s disease and multiple sclerosis are characterised by oxidative stress, Nrf2 is commonly activated in these diseases. Evidence demonstrates that Nrf2 activity is repressed in neurons in vitro, and only cultured astrocytes respond strongly to Nrf2 inducers, leading to the interpretation that Nrf2 signalling is largely restricted to astrocytes. However, Nrf2 activity can be observed in neurons in post-mortem brain tissue and animal models of disease. Thus this interpretation may be false, and a detailed analysis of the cell type expression of Nrf2 in neurodegenerative diseases is required. This review describes the evidence for Nrf2 activation in each cell type in prominent neurodegenerative diseases and normal aging in human brain and animal models of neurodegeneration, the response to pharmacological and genetic modulation of Nrf2, and clinical trials involving Nrf2-modifying drugs.

## 1. Introduction

Neurodegenerative diseases such as Alzheimer’s disease, Parkinson’s disease, amyotrophic lateral sclerosis, Huntington’s disease and multiple sclerosis are characterised by the failure of specific populations of neurons, such as hippocampal neurons in Alzheimer’s disease, dopaminergic neurons in the substantia nigra in Parkinson’s disease, motor neurons in the spinal cord and motor cortex in amyotrophic lateral sclerosis, and striatal medium spiny neurons in Huntington’s disease. The failure of each of these specific neuronal populations is governed by disease-dependent factors that remain unclear. However, several consistent features are common to all of these diseases, including oxidative stress and glial activation, in which astrocytes gain a reactive phenotype that can be either protective or detrimental to neurons [1]. This review will discuss the evidence for activation of nuclear factor erythroid 2-related factor 2 (Nrf2) in neurodegeneration, pharmacological and genetic targeting of Nrf2, and in which cells Nrf2 is activated, focusing on Alzheimer’s disease, Parkinson’s disease, amyotrophic lateral sclerosis, Huntington’s disease and multiple sclerosis. Nrf2 is also involved in stroke, which has been recently discussed in other excellent reviews [2,3].

## 2. Mechanisms of Nrf2 Activation and Regulation

**Figure 1:**
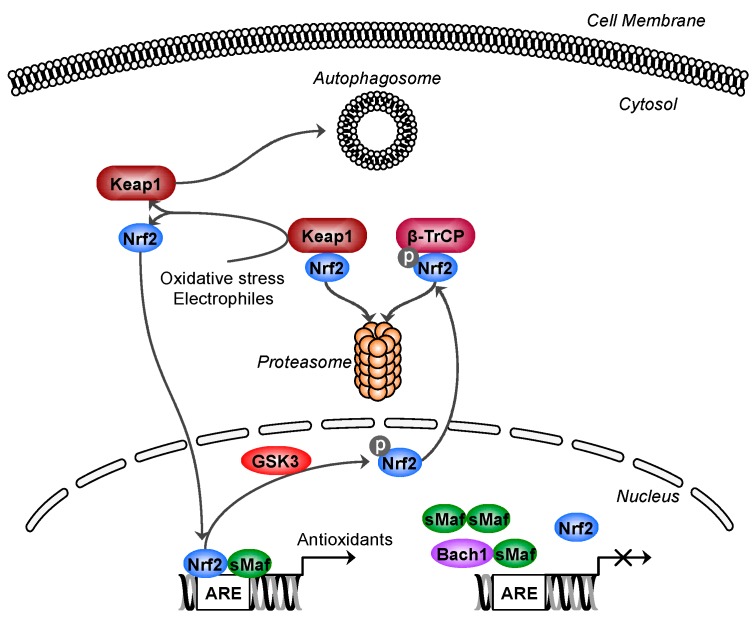
Mechanisms of nuclear factor erythroid 2-related factor 2 (Nrf2) regulation. Under normal conditions, Nrf2 is bound in the cytosol by its negative regulator, kelch-like ECH-associated protein 1 (Keap1), and constitutively targeted for proteasomal degradation. Exposure to oxidative stress or electrophiles causes Nrf2 to dissociate from Keap1 (which is degraded by autophagy), allowing Nrf2 to translocate to the nucleus, where it heterodimerises with small Maf proteins (sMaf) in order to bind to antioxidant response elements (ARE) in the promoter region of antioxidant target genes. The availability of sMaf regulates Nrf2 binding to DNA, and BTB and CNC homology 1 (Bach1) negatively regulates Nrf2 binding to DNA by competing with Nrf2 for heterodimerisation with sMaf. Phosphorylation of Nrf2 by glycogen synthase kinase 3 (GSK3) promotes its nuclear export and proteasomal degradation facilitated by β-transducin repeat-containing protein (βTrCP).

Nrf2 is a transcription factor regulating the transcription of a battery of antioxidant genes. Under normal conditions, Nrf2 is bound in the cytosol by its negative regulator Kelch-like erythroid cell-derived protein with cap ‘n’ collar (CNC) homology associated protein 1 (Keap1) (Figure 1). Keap1 is a substrate adapter protein for the Cullin3-Rbx1 E3 ligase complex and constitutively targets Nrf2 for proteasomal degradation. Modification of sensitive cysteine residues on Keap1 by oxidative stress or electrophiles causes Nrf2 to dissociate from Keap1, the latter then degraded by autophagy [4]. This stabilises Nrf2, allowing it to translocate to the nucleus where it binds to antioxidant response elements (ARE) in the promoter region of target genes. DNA binding requires heterodimerisation with small Maf proteins, and the availability of these small Maf proteins regulates Nrf2 activity [5,6]. Broad-complex, tramtrack, and bric-a-brac (BTB) and CNC homology 1 (Bach1) negatively regulates nuclear Nrf2 activity by competing with Nrf2 for binding to small Maf proteins [7]. In addition to Keap1, Nrf2 can also be bound by β-transducin repeat-containing protein, which is substrate adapter protein for the Skp1-Cullin1 E3 ligase and targets Nrf2 for proteasomal degradation in a manner dependent on Nrf2 phosphorylation by glycogen synthase kinase 3 (GSK3) [8] (Figure 1). Classical Nrf2 target genes encode antioxidant proteins, including NAD(P)H quinone dehydrogenase (NQO1), heme oxygenase 1 (HO1) and the two subunits of glutamate-cysteine ligase (GCL), GCLC and GCLM, which is the rate-limiting enzyme for glutathione synthesis. Nrf2 also regulates several genes involved in autophagy including SQSTM1, encoding the p62 protein [9], and many genes encoding antioxidant proteins such as glutathione peroxidases, glutathione-*S*-transferases, peroxiredoxins, thioredoxins, thioredoxin reductases, and NADPH regenerating enzymes [10]. Nrf2 and Keap1, the molecular mechanisms for activation and gene targets, are highly conserved across mammals and zebrafish, while drosophila and *C. elegans* express Nrf2 homologs CNC isoform C (CncC) and skinhead-1, respectively [11,12]. Nrf2 is dispensable under normal conditions, and its deletion does not greatly alter basal expression of its target genes [13,14,15]. Thus, Nrf2 responds to stress to upregulate protective genes. 

## 3. Nrf2 in Normal Aging

Nrf2 and ARE signalling are altered with age. Evidence for altered expression of Nrf2 target genes with aging is mixed [16], whereas Nrf2 activity is clearly impaired with age in rat liver [17]. In contrast, compiling data from several independent studies reveals that ARE–human placental alkaline phosphatase (hPAP) reporter mice appear to exhibit increasing Nrf2 activity with age in the dorsal horn of the spinal cord; while little ARE activity is present in 60 day old mice [18], strong ARE activity is evident in 110 day old mice [19], and is very intense in 6 month old ARE–hPAP mice [20]. ARE activity also appears in the ventral horn of the spinal cord of these mice with age, albeit less intensely [19,20]. This is consistent with increased Nrf2 protein in spinal cord of aged mice [21], and increased Nrf2 activity in cerebellum of aged mice [22]. In contrast to the spinal cord and cerebellum, Nrf2 and Nrf2 target gene expression are decreased in substantia nigra, ventral tegmental area and hippocampus of aged rats [23]. Interestingly, in young ARE–hPAP mice, ARE activity appears to be greatest in the substantia nigra, striatum and ventral hippocampus [24,25]. Hence it appears that the Nrf2 response with age is region-dependent, and that aging is associated with a loss of Nrf2 activity particularly from regions that have high Nrf2 activity in young animals. There has been little analysis of Nrf2 activity in aging by cell type. Although spinal cord ARE activity is present in multiple cell types [19,20], the elevated Nrf2 protein evident in spinal cord of aged mice is largely restricted to astrocytes [21]. 

Aging alters the capacity of Nrf2 to respond to insults. The Nrf2 response to insult is impaired in rhesus macaque vascular smooth muscle in aged compared to younger animals [26]. Similarly, response of the drosophila Nrf2 homolog CncC to acute insult is impaired in old drosophila compared to younger flies [27]. The influence of aging on Nrf2 function has also been assessed in brain. Exposure to airborne pollution elicits a robust Nrf2 response in the cerebellum of young mice, while the same treatment in aged mice has no effect on Nrf2 signalling [22]. Nrf2 response is also region-dependent, whereby treatment of aged rats with testosterone restores Nrf2 function in the basal ganglia but not hippocampus, and is associated with improved exploratory and motor behavior, and dopaminergic cell numbers [23]. Unfortunately, the effect of testosterone on young rats was not investigated in this study. These studies suggest that the capacity of Nrf2 to respond to insults is altered with age, and in a region-dependent manner.

Deletion of Nrf2 causes widespread transcriptomic changes that mirror those of aging. Transcriptomic analyses reveal that of the eleven functional pathways altered in Nrf2^−/−^ mice, seven correspond to pathways that are dysregulated in human aging [15], suggesting that Nrf2 may be impaired with age. Furthermore, deletion of Nrf2 causes the appearance of spongiform leukoencephalopathy, myelin disruption and widespread astrogliosis in aged mice [28]. This is not accompanied by any apparent neuronal damage or microglial activation [28], suggesting Nrf2 is important for astrocyte and oligodendrocyte function with age. In six-month old mice, Nrf2 deletion does not detectably alter performance on learning and memory behavioural tests, but does impair hippocampal long-term potentiation [15]. These studies indicate that loss of Nrf2 influences both glial and neuronal activity. However, it is not clear whether a given cell type is directly or indirectly affected. 

## 4. Parkinson’s Disease

Parkinson’s disease is the second most common neurodegenerative disease after Alzheimer’s disease. Clinical symptoms include progressive motor disturbances such as bradykinesia, muscular rigidity and resting tremor, and non-motor features such as olfactory, cognitive, psychiatric and autonomic disruptions. Neuropathologically, Parkinson’s disease primarily involves the loss of dopaminergic neurons from the substantia nigra, and appearance of Lewy bodies containing aggregated α-synuclein.

A consistent feature of post-mortem Parkinson’s disease brain is a substantial loss of glutathione from the substantia nigra [29,30,31,32,33,34], and the presence of oxidative stress [35]. Both are well known to induce Nrf2 activity [13]. Accordingly, analysis of substantia nigra neurons of post-mortem Parkinson’s disease brain reveals robust accumulation of nuclear Nrf2 as compared to normal brain [36], and aberrant localisation of Keap1 to Lewy bodies [37]. 

Expression of Nrf2 target proteins are also altered in Parkinson’s disease. NQO1 and HO1 are strongly elevated in Parkinson’s disease brain, an effect restricted to the substantia nigra pars compacta compared to adjacent tissue [38,39]. Peroxiredoxin 6 expression is also greatly increased in Parkinson’s disease substantia nigra [40], as is glutathione peroxidase 1 in cingulate gyrus and middle frontal gyrus (substantia nigra was not investigated) [41]. Another Nrf2-regulated protein, γ-glutamyl transferase, is also increased in the substantia nigra of Parkinson’s disease brains [30]. This enzyme is involved in the extracellular degradation of glutathione exported by astrocytes and important for supplying glutathione precursors to neurons [42]. 

However, further findings are not consistent with Nrf2 activation. The activities of glutathione-*S*-transferase, glutathione peroxidase and GCL are unchanged in post-mortem Parkinson’s disease brain [30]. Ferritin, while primarily regulated by iron regulatory protein signalling, is also upregulated by Nrf2 activity. However, there is no clear change in ferritin levels in Parkinson’s disease substantia nigra, with levels variously reported as increased [43], decreased [44,45] or unchanged [46]. Proteomic studies find conflicting results for many Nrf2 targets [47,48]. A potential contributing factor for these inconsistent results is that some of these proteins can be regulated by additional mechanisms other than Nrf2. Additionally, the obvious caveats of post-mortem studies should be kept in mind. Overall, findings from human post-mortem analyses are generally consistent with upregulation of Nrf2 activity in Parkinson’s disease brain, particularly the substantia nigra, the primary site of dopaminergic neuron loss.

Dopaminergic neurons derived from familial Parkinson’s disease patient induced pluripotent stem cells (iPSC) harbour disease-causing mutations. These cells exhibit elevated basal oxidative stress, mitochondrial abnormalities, decreased glutathione levels and elevated Nrf2 activation [49,50]. Furthermore, these cells are more susceptible to oxidative stress [50]. Similarly, Parkinson’s disease patient olfactory neurosphere-derived cells display oxidative stress, decreased glutathione and decreased NQO1 expression, even though Nrf2 levels are maintained, and exhibit an altered response to stimulation by sulforaphane compared to controls [51]. 

In mice, Nrf2 activity is strongly increased in the substantia nigra in response to Parkinson’s disease-related toxins including 1-methyl-4-phenyl-1,2,3,6-tetrahydropyridine (MPTP) [24,52,53,54], 6-hydroxydopamine [55] or mutated α-synuclein [20], although rotenone decreases Nrf2 activity in rats [56]. Furthermore, exposing Nrf2^−/−^ mice to MPTP, 6-hydroxydopamine or α-synuclein causes more severe loss of dopaminergic neurons from substantia nigra and striatum compared to wild-type mice [24,54,57,58,59], clearly indicating the role of Nrf2 in dopaminergic neuronal survival. Together these results indicate that Nrf2 is activated in animal models of, and in human Parkinson’s disease, but this activation is clearly insufficient to completely prevent neuronal failure.

Although endogenous expression of Nrf2 is unable to prevent cell death, pharmacological activation of Nrf2 prevents neuronal loss in animal models of Parkinson’s disease (Table 1). Sulforaphane is a small-molecule activator of Nrf2 able to cross the blood–brain barrier [60]. Administration of sulforaphane to mice prevents the toxicity of MPTP [60], 6-hydroxydopamine [61] and rotenone [62], and prevents α-synuclein toxicity in drosophila [63]. Dimethyl fumarate, which is clinically approved for the treatment of multiple sclerosis [64,65], is also effective in MPTP, 6-hydroxydopamine and α-synuclein models of Parkinson’s disease [13,66,67,68]. Many other Nrf2-inducing drugs are protective in animal models of Parkinson’s disease including triterpenoids (2-cyano-3-,12-dioxooleana-1,9(11)-dien-28-oic acid (CDDO) derivatives) [69,70], curcumin [56,71], 3H-1,2-dithiole-3-thione [72], carnosic acid [73] and resveratrol [74,75,76,77] (see [2] for further compounds). Cu^II^(atsm) provides protection in multiple mouse models of Parkinson’s disease [78], and has recently be shown to activate Nrf2 in mice [79]. Cu^II^(atsm) is currently under investigation in a phase 1 clinical trial in Parkinson’s disease patients, including efficacy secondary outcome measures (NCT03204929; Table 2). The requirement for Nrf2 in the protective effect of these Nrf2-inducing compounds has been demonstrated for sulforaphane, dimethyl fumarate, triterpenoids and 3H-1,2-dithiole-3-thione [13,60,67,69,72], whereby their protective capacity is diminished in Nrf2^−/−^ mice (Table 1). Furthermore, some Parkinson’s disease drugs are able to activate Nrf2, including deprenyl and apomorphine [80,81,82]. Lastly, knockdown of Keap1, which increases Nrf2, provides partial protection against MPTP toxicity in mice [83].

### Cell Type-Specific Activation of Nrf2 in Parkinson’s Disease

The increase in nuclear Nrf2 evident in the substantia nigra of human post-mortem Parkinson’s disease brain (as compared to normal brain) appears to be restricted to neurons [36]. In contrast, NQO1 [38] and peroxiredoxin 6 [40] are strongly expressed in astrocytes in substantia nigra of Parkinson’s disease brain, with more infrequent expression in neurons. Similarly, while HO1 is mildly expressed in normal and Parkinson’s disease neurons, HO1 expression is greatly increased in Parkinson’s disease astrocytes, specific to the substantia nigra compared to other regions [39]. Increased γ-glutamyl transferase [30] is likely to be limited to astrocytes as this enzyme is involved in the extracellular degradation of glutathione exported by astrocytes and important for supplying glutathione precursors to neurons [42]. A histochemical study showed a profound loss of glutathione from surviving dopaminergic neurons in Parkinson’s disease substantia nigra, while glutathione was maintained in glia [33]. While Nrf2 activation in vivo and in vitro can have profound effects on microglia [58,67], and Nrf2 activation can be observed in cultured microglia [58,59,99], direct evidence for Nrf2 activation specifically in microglia in vivo is limited to elevated GPx1 in the cingulate gyrus [41] and HO1 in the substantia nigra of post-mortem human Parkinson’s disease brains [59]. Nrf2 activation in oligodendrocytes in the context of Parkinson’s disease has not been reported. Together these findings indicate that the upregulation of Nrf2 target genes is largely restricted to astrocytes in Parkinson’s disease brain.

In normal mice, increased expression of HO1 concomitant with Nrf2 induction is restricted to astrocytes, sometimes microglia, and not expressed in neurons [60,85], consistent with HO1 expression in human brain [39,59], suggesting HO1 is upregulated exclusively in glia. Furthermore, in the basal ganglia of MPTP-treated mice, astrocytes are the main cell type to upregulate Nrf2 in response to sulforaphane treatment [60]. In contrast, the increased Nrf2 expression evident in response to dimethyl fumarate in MPTP-treated mice is at least partially localised to midbrain neurons [66]. Hence it appears that Nrf2 inducers may differentially target certain cell populations. Cell-specific Nrf2 activation for other Nrf2 inducers has not been assessed.

Further evidence for the importance of astrocytic Nrf2 expression comes from overexpression experiments (Table 1). Nrf2 overexpressed on the GFAP promoter thus restricting overexpression to astrocytes renders mice resistant to MPTP toxicity, with not as much loss of striatal tyrosine hydroxylase and less gliosis [24]. Overexpression of Nrf2 in astrocytes also protects mice from mutant α-synuclein [20], and transplanting Nrf2-overexpressing astrocytes into mouse striatum protects against 6-hydroxydopamine toxicity [57]. Furthermore, crossing mice overexpressing Nrf2 in astrocytes with Nrf2^−/−^ mice, such that astrocytes are the only cells expressing Nrf2 is also protective against MPTP toxicity [24]. This indicates that neuronal Nrf2 is not explicitly required to prevent neuronal death. Furthermore, these results indicate astrocytes are key mediators of Nrf2 signalling. It remains to be determined whether activation of Nrf2 specifically in neurons is similarly protective in Parkinson’s disease models.

## 5. Alzheimer’s Disease

Alzheimer’s disease is the most common form of dementia and most prevalent neurodegenerative disease. It involves the progressive age-related accumulation of extracellular plaques containing amyloid-β and intracellular neurofibrillary tangles containing tau, and synaptic dysfunction and neuron loss. Neuropathology begins in the hippocampus and temporal cortex and progresses to other brain regions, leading to cognitive and memory deficits.

Analysis of human post-mortem brain tissue reveals that Nrf2 appears to be abundant in the nuclei and weakly expressed in cytosol of neurons from normal brain [36]. However, in Alzheimer’s disease brain, Nrf2 is highly excluded from nuclei of neurons and strongly cytosolic [36] (Table 3). This is apparent in immunohistochemical staining of hippocampal neurons and western blot of nuclear fractions from frontal cortex homogenates [36], suggesting Nrf2 activity is decreased in Alzheimer’s disease. However, more recent studies investigating post-mortem Alzheimer’s disease brain tissue found an increase in Nrf2 and p62 in cells expressing high levels of amyloid precursor protein or neurofibrillary tangles, and other Nrf2 targets are also elevated [9,100], as are Nrf2 target gene transcripts [37]. Keap1 is also dysregulated, found in neurofibrillary tangles associated with p62 [37]. In contrast to the earlier findings [36], these studies suggest Nrf2 signalling is elevated in Alzheimer’s disease.

Supporting increased Nrf2 activity in Alzheimer’s disease, further analysis of Nrf2 targets in post-mortem Alzheimer’s disease brain shows increased NQO1 expression in hippocampus [104,105] and frontal cortex (although less strongly than in hippocampus) [109], while HO1 is increased in temporal cortex and hippocampus of Alzheimer’s disease brains [9,100,101,102,103]. Expression of other genes regulated by Nrf2 is less consistent (Table 3). Overall, the evidence indicates that Nrf2 activity is more often elevated in hippocampus and temporal cortex in Alzheimer’s disease, whereas Nrf2 activity in frontal cortex is more variable. Hence it appears that there are regional variations in Nrf2 activation in Alzheimer’s disease, with greater activity in the regions where pathology is most severe.

In mice, Nrf2 expression is decreased in hippocampal neurons and frontal cortex homogenates of 16-month-old amyloid precursor protein/presenilin-1 (APP/PS1) mice compared to wild-type controls [112]. Decreased mRNA expression of Nrf2 targets is apparent in six-month old APP/PS1 frontal cortex homogenates, coinciding with the appearance of amyloid-β pathology [112]. However, a more recent report found that Nrf2 and its targets are either unchanged or increased in the hippocampus of the same Alzheimer’s disease model mice at a similar age [14]. Furthermore, in an amyloid-β rat model of Alzheimer’s disease, Nrf2 signalling is decreased in cortex but not hippocampus [113], while another study utilising the same model found elevated Nrf2 in hippocampus [114]. Finally, hippocampal expression of mutated tau elevates HO1 and GCLC transcripts in wild-type but not Nrf2^−/−^ mice [100]. These results somewhat mirror those in the human Alzheimer’s disease brain, with a tendency for greater Nrf2 activity in hippocampus and less activity in frontal cortex.

In addition to regional variations, age-dependent changes in Nrf2 activity could contribute to inconsistent results. Three studies have examined aspects of Nrf2 signalling in the 3xTg Alzheimer’s disease mouse model and together these studies indicate biphasic age-dependent changes in Nrf2 activity. In early adulthood (2–3 months of age), Nrf2 activity is elevated in these mice compared to controls, with increased Nrf2 and NQO1 expression [115,116] (although GCLC is unchanged and HO1 and peroxiredoxin 1 expression are decreased [116]). But then Nrf2 and NQO1 expression quickly decline with age, with similar NQO1 expression as controls from 4–6 months of age [115] and lower Nrf2 expression in Alzheimer’s disease mice from 11–21 months of age [116,117]. 

Although Nrf2 activity varies in region and age-dependent manners, it is clear that Nrf2 activity is insufficient to prevent oxidative stress and neuronal cell dysfunction in Alzheimer’s disease. Supporting this, transcriptomic analyses reveal that Nrf2 deletion in mice causes alterations in functional pathways recapitulating those altered in Alzheimer’s disease [15]. That a lack of Nrf2 mirrors Alzheimer’s disease suggests that Nrf2 activity is impaired or insufficient in the disease. However, results from direct modulation of Nrf2 in mouse models clearly demonstrate the protective influence of Nrf2 in Alzheimer’s disease. Nrf2^−/−^ mice crossed with APP/PS1 or mutant HsAPP^V717I^/HsMAPT^P301L^ mice exhibit exacerbated astrocyte and microglia activation, and worsened amyloid-β pathology [9,14], as well as tau pathology and impaired learning and memory [15], whereas overexpression of Nrf2 in hippocampal neurons via lentiviral transduction lessens astrocyte activation and improves cognition without diminishing amyloid-β pathology [118]. 

Pharmacological activation of Nrf2 is protective in various animal models of Alzheimer’s disease (Table 4). For example, the highly potent triterpenoid CDDO-methylamide improves cognition and abrogates amyloid-β pathology in Tg19959 mice [119], while dl-3-n-butylphthalide is similarly protective in APP/PS1 mice [120]. Methysticin improves cognition and ameliorates gliosis in APP/PS1 mice [121], and carnosic acid is protective in multiple Alzheimer’s disease mouse models [122]. Nrf2 activation by arsenite or sulforaphane prevents toxicity associated with amyloid-β administration to rats and mice [114,123]. More recently, alternative treatments are being investigated, such as direct inhibition of Keap1, which induces Nrf2 activity and prevents amyloid-β toxicity in drosophila [124]. Importantly, none of these studies determined the requirement of Nrf2 for the protective effects of the compounds investigated. Curcumin and resveratrol have been extensively studied in preclinical animal models of Alzheimer’s disease (Table 4). While Nrf2 activation was not considered as a mechanism of action in these studies, Nrf2 activation by curcumin and resveratrol has been extensively demonstrated elsewhere [125,126].

Considerable evidence indicates GSK3 is involved in the pathogenesis of Alzheimer’s disease [144]. GSK3 is a negative regulator of Nrf2 [8], and inhibition of GSK3 promotes Nrf2 activity [145,146]. Pyrrolidine dithiocarbamate, which induces Nrf2 [147] and inhibits GSK3 [142,147], is protective in APP/PS1 mice [142]. Other studies show that although inhibition of GSK3 is protective in models of Alzheimer’s disease [146,148], this effect can occur independently of Nrf2 activation [124,149]. New approaches such as GSK3 inhibitor/Nrf2 inducer dual function drugs are being developed [149]. 

Clinical trials with Nrf2 inducing agents such as resveratrol and curcumin in Alzheimer’s disease patients have produced limited results [86,88], presumably due to poor bioavailability and brain penetrance of the compounds [125], although a retrospective subset analysis found positive results for resveratrol on Alzheimer’s Disease Cooperative Study—Activities of Daily Living (ADCS-ADL) clinical scores [89] (Table 2). dl-3-n-butylphthalide has generated positive outcomes in a phase 2 clinical trial in Chinese patients with vascular cognitive impairment without dementia [150], a population at high risk of developing dementia [151], and is currently under investigation in Alzheimer’s disease patients (NCT02711683; Table 2).

### Cell Type-Specific Activation of Nrf2 in Alzheimer’s Disease

As opposed to Parkinson’s disease, evidence for Nrf2 activation in specific cell types in Alzheimer’s disease is limited. In post-mortem Alzheimer’s disease brain, while Nrf2 has been shown to be both elevated and decreased hippocampal neurons [9,36], Nrf2 is also observed in the nucleus of some hippocampal astrocytes [36]. Although increased NQO1 is evident in hippocampal and frontal cortex neurons in Alzheimer’s disease brain [104,105,109], NQO1 is strongly increased in astrocytes near plaques in both regions [105,109]. HO1 is increased in both neurons and astrocytes in the temporal cortex and hippocampus [101,102,103], and in microglia in the hippocampus [100] of Alzheimer’s disease brains. HO1 is also evident in astrocytes and microglia in response to mutated tau expression in the hippocampus of mice [100].

Overexpression studies do not directly indicate which cell types are responsible for the endogenous Nrf2 response, but they do provide evidence for which cells may be involved. Overexpression of Nrf2 specifically in hippocampal neurons alleviates cognitive dysfunction in APP/PS1 mice [118] suggesting that Nrf2 activation in neurons is sufficient to prevent neuronal dysfunction in Alzheimer’s disease. Overexpression of Nrf2 in astrocytes has not been investigated in Alzheimer’s disease models, and therefore it is unclear whether activation of Nrf2 specifically in astrocytes is equally effective in alleviating Alzheimer’s disease symptoms.

Overall, the available data indicate Nrf2 activity is increased in neurons, astrocytes and possibly microglia in Alzheimer’s disease, while oligodendrocytes have not been studied. However, the cell type-specific requirement of Nrf2 expression in Alzheimer’s disease is unknown. Furthermore, the cell type/s targeted by pharmacological inducers of Nrf2 have not been assessed in the context of Alzheimer’s disease.

## 6. Amyotrophic Lateral Sclerosis

Amyotrophic lateral sclerosis is the most common motor neuron disease. It involves the progressive failure of motor neurons in the spinal cord and motor cortex, causing progressive paralysis inexorably leading to death usually within 3–5 years of diagnosis. Familial amyotrophic lateral sclerosis can be caused by mutations in superoxide dismutase 1 (SOD1), transactive response DNA-binding protein 43 (TDP-43) and several other genes. Sporadic disease is most often accompanied by pathological inclusions containing TDP-43 [152].

Nrf2 mRNA and protein is decreased in post-mortem amyotrophic lateral sclerosis motor cortex and spinal cord compared to normal brain [153]. As in Alzheimer’s disease, although expression of Keap1 is unchanged [153], Keap1 is found associated with intracellular inclusions in spinal cord motor neurons [37], and Keap1 mRNA is elevated in the motor cortex of post-mortem amyotrophic lateral sclerosis brain [153]. Motor neurons cultured from SOD1-G93A amyotrophic lateral sclerosis model mice or neuroblastoma spinal cord (NSC)34 motor neuron-like cells expressing mutated SOD1 have diminished Nrf2 activity [154,155], and transcriptomic analysis of NSC34 cells expressing mutated SOD1 show down-regulated Nrf2 and Nrf2 target genes [156]. Hence, Nrf2 appears to be diminished in amyotrophic lateral sclerosis, particularly in motor neurons.

In contrast to human tissue and cultured cells, Nrf2 activity is consistently elevated in the spinal cord of SOD1-G93A rodent models of amyotrophic lateral sclerosis [19,157,158,159]. Regional analysis of SOD1-G93A mice crossed with ARE–hPAP reporter mice reveals Nrf2 activity is minimal in motor cortex, present in spinal cord, and intense in skeletal muscle [19]. Nrf2 signalling in skeletal muscle is evident from before motor symptom onset [19]. That Nrf2 signalling is greater in skeletal muscle compared to spinal cord is consistent with the dying back hypothesis of motor neuron degeneration [160], which posits motor neuron dysfunction is initiated at neuromuscular junctions where their presynaptic terminals contact muscle fibers.

Interestingly, deletion of Nrf2 does not greatly alter disease progression in mice modeling amyotrophic lateral sclerosis. Nrf2^−/−^ mice crossed with SOD1-G93A, SOD1-G85R or SOD1-H46R mice have either unchanged or only mildly altered symptom onset and survival [159,161,162]. Surprisingly, Guo et al. report that crossing Nrf2^−/−^ mice with SOD-G93A mice does not alter the elevated expression of Nrf2 targets HO1, GCLM and GCLC, although NQO1 elevation is blocked [159]. These results suggest that some classical Nrf2 target genes can be regulated independently of Nrf2 in amyotrophic lateral sclerosis mice. 

That Nrf2 deletion has little impact on disease phenotype while Nrf2 is elevated in animal models suggests that Nrf2 signalling is impaired in amyotrophic lateral sclerosis. Recent evidence suggests a mechanism involving disrupted TDP-43 function. Fibroblasts from amyotrophic lateral sclerosis patients harbouring mutated TDP-43 have disrupted Nrf2 signalling, where basal Nrf2 activity is elevated but their response to the Nrf2 inducer arsenite [114] is impaired [163]. Interestingly, Nrf2 protein and Nrf2 target gene transcripts are increased in these cells, but the transcripts do not appear to be effectively translated into protein [163]. Heterogenous ribonucleoproteins (hnRNPs) such as TDP-43 and hnRNP K are RNA binding proteins, and are involved in trafficking mRNA [164]. Mutant TDP-43-dependent mislocalised hnRNP K co-precipitates with Nrf2 and glutathione peroxidase 1 mRNA, suggesting hnRNPs are aberrantly binding mRNA of antioxidant genes, preventing their translation [163]. Consistent with these findings, NSC34 cells expressing mutated TDP-43 have elevated Nrf2 but decreased NQO1 protein [165], and decreased HO1 protein [166], again suggesting translation of Nrf2 activity into antioxidant proteins is impaired. However, as opposed to robust accumulation of Nrf2 following arsenite [163], sulforaphane does not cause Nrf2 accumulation or signalling in NSC34 cells expressing mutant TDP-43 [166]. As TDP-43 pathology is highly prevalent in amyotrophic lateral sclerosis [167], these results suggest that Nrf2 signalling may be disrupted in this manner in the majority of amyotrophic lateral sclerosis cases.

Although endogenous Nrf2 signalling appears to be impaired in amyotrophic lateral sclerosis, the role of Nrf2 in preventing motor neuron death is clearly demonstrated by experiments pharmacologically targeting Nrf2. Various agents activating Nrf2 have been assessed in animal models of amyotrophic lateral sclerosis (Table 5). Highly potent synthetic triterpenoids exhibit efficacy in SOD1-G93A mice, extending survival and activating Nrf2 genes in spinal cord [168]. dl-3-n-butylphthalide similarly extends survival of SOD1-G93A mice and activates Nrf2 target genes and ameliorates gliosis in spinal cord [169]. Although initially determined to have no positive effect [170], multiple subsequent studies have shown efficacy of resveratrol in SOD1-G93A mice [171,172,173]. In silico screening of over 3 million compounds for antioxidative capacity identified *N*-(4-(2-pyridyl)(1,3-thiazol-2-yl))-2-(2,4,6-trimethylphenoxy) acetamide (CPN-9), which activates Nrf2 and extends the survival of SOD1-H46R mice even when treated post-symptom onset [174]. A subsequent study utilising in silico drug design based on CPN-9 generated the novel compound WN1316, which also activates Nrf2 and extends survival of SOD1-H46R and SOD1-G93A mice and decreases gliosis in the spinal cord [175]. The receptor-inactive S[+] enantiomer of the Parkinson’s disease drug apomorphine was identified from a screen of compounds for Nrf2 activation and brain penetrance [176]. Treatment with S[+]-apomorphine slightly improves the motor function of SOD1-G93A mice but did not extend survival [176]. The Nrf2 inducing compound pyrrolidine dithiocarbamate [147], while protective in Alzheimer’s disease [142], worsens phenotype in SOD1-G93A mice [177]. One possible explanation for this surprising result is that pyrrolidine dithiocarbamate inhibits the immunoproteasome [177], which is elevated in these mice [177,178]. Immunoproteasome function may be beneficial in these mice, and its inhibition by pyrrolidine dithiocarbamate may outweigh any protective gains made by Nrf2 induction. Finally, lentiviral delivery of Nrf2 in combination with excitatory amino acid transporter 2 (EAAT2) and glutamate dehydrogenase 2 (GDH2) to SOD1-G93A mice is protective, but not Nrf2 in isolation [179].

Edaravone, a free radical scavenger, is the first new drug to be approved for the treatment of amyotrophic lateral sclerosis since Riluzole in 1995 [187]. Although two phase 3 clinical trials found no efficacy [91,92], a third phase 3 trial showed efficacy in a specific population of amyotrophic lateral sclerosis patients with short disease duration and mild symptoms [90] (Table 2). Although the primary action of the drug is a free radical scavenger, edaravone has been shown to activate Nrf2 in PC12 cells [188] and in rats co-treated with kainate [189]; hence, Nrf2 activation may contribute to its mechanism of action. 

Cu^II^(atsm) improves motor function and extends survival in multiple mouse models of amyotrophic lateral sclerosis, including when administered post-symptom onset [180,181,182,183,184,185], and has recently been shown to activate Nrf2 in mice [79]. Cu^II^(atsm) is currently being investigated in a phase 1 clinical trial (NCT02870634), and a linked phase 1/2 extension trial (NCT03136809) in amyotrophic lateral sclerosis patients, both with efficacy secondary outcome measures (Table 2).

### Cell Type-Specific Activation of Nrf2 in Amyotrophic Lateral Sclerosis

Although Nrf2 expression in post-mortem normal human motor cortex and spinal cord is mainly neuronal, and is diminished in amyotrophic lateral sclerosis, Nrf2 is evident in astrocytes of human amyotrophic lateral sclerosis motor cortex [153]. This result is analogous to the appearance of Nrf2 in activated astrocytes in Alzheimer’s disease brain [36]. This effect is more pronounced in mouse models of amyotrophic lateral sclerosis. Although elevated Nrf2 is apparent in disease-model neurons, Nrf2 activation is stronger in spinal cord astrocytes with Nrf2 targets upregulated specifically in activated astrocytes [19,157,158]. Hence, elevated Nrf2 coincides with astrocyte activation in human and mouse spinal cord in amyotrophic lateral sclerosis. Nrf2 expression in microglia and oligodendrocytes has not been assessed in amyotrophic lateral sclerosis.

The cell types targeted by pharmacological Nrf2 inducers have not been assessed in the context of amyotrophic lateral sclerosis. However, overexpression studies provide strong evidence for the key role of astrocyte Nrf2 activation in amyotrophic lateral sclerosis (Table 5). Viral delivery of Nrf2 via intramuscular injections does not alter the phenotype of SOD1-G93A mice [186]. Similarly, Nrf2 overexpression targeted to skeletal muscle delays onset but does not alter survival of SOD1-G93A mice [161]. Overexpression of Nrf2 specifically in motor neurons also moderately delays symptom onset, but has no effect on survival [161]. In contrast, overexpression of Nrf2 specifically in astrocytes significantly delays symptom onset and extends the survival of SOD1-G93A mice [18]. Hence, as opposed to Alzheimer’s disease, where neuronal overexpression of Nrf2 is protective [118], overexpression of Nrf2 is only strongly protective in amyotrophic lateral sclerosis when targeted to astrocytes.

Lack of protection by neuronal Nrf2 overexpression in amyotrophic lateral sclerosis is consistent with evidence that, for this disease in particular, the activity of astrocytes is especially important for neuronal survival. In co-culture experiments, spinal cord astrocytes from amyotrophic lateral sclerosis patients or mice kill co-cultured motor neurons [190,191]. Nrf2 overexpression in neurons [18] does not appear to be sufficient to overcome the negative influence of astrocytes. In contrast, Nrf2 activation prevents astrocyte killing of co-cultured motor neurons in a glutathione dependent mechanism [18,157,192]. These results demonstrate that astrocytic Nrf2 is required to prevent astrocyte-dependent damage to neurons, and highlights the importance of astrocyte-dependent Nrf2 expression for preventing neurodegeneration. 

## 7. Huntington’s Disease

Huntington’s disease is a relatively rare (5–10 per 100,000) autosomal-dominant neurodegenerative disease. It is caused by poly-glutamine expansion in the huntingtin gene, giving rise to aggregation-prone huntingtin protein. Neuropathology involves failure of medium-sized spiny neurons in the striatum, and clinical symptoms include chorea and rigidity, as well as dementia and other cognitive and neuropsychiatric symptoms. Mitochondrial complex II inhibitors such as 3-nitropropionic acid and malonate effectively recapitulate several features of Huntington’s disease, including striatal degeneration and disruption of motor function.

Nrf2 has not been well studied in human Huntington’s disease tissue. While there has been no assessment of Nrf2 expression, some Nrf2 targets have been assessed in human post-mortem Huntington’s disease brain. HO1 is elevated [193] and glutathione synthetase is strongly decreased in striatum [194], while striatal NQO1 and cortical glutathione synthetase and NQO1 are unchanged [194]. No alterations in glutathione content have been detected in cortex, striatum or substantia nigra in human post-mortem Huntington’s disease brain [31].

In blood from Huntington’s disease patients, glutathione peroxidase activity has been reported as both increased and decreased in erythrocytes [195,196], while glutathione reductase activity is decreased [195]. Plasma glutathione levels have been reported as decreased or unchanged [195,197]. 

Evidence for Nrf2 activity in genetic models of Huntington’s disease is mixed. In the R6/2 model, Nrf2 targets are elevated in striatum [198], whereas striatal Nrf2 mRNA is decreased [199], and thioredoxin reductase 3 mRNA and plasma glutathione are increased in N171-82Q mice [200]. Brain glutathione is unchanged in YAC128 mice [201], and glutathione peroxidase activity is unchanged in R6/1 mice [202]. 

Together, these studies show that Nrf2 is not greatly altered in Huntington’s disease, in human brain or most genetic animal models. That Nrf2 is not induced despite obvious oxidative stress [203] suggests that Nrf2 signalling is impaired in Huntington’s disease. Indeed, in cultured cells, Huntington’s disease patient iPSC-derived neural stem cells exhibit impaired capacity for pharmacological Nrf2 induction [84]. Similarly, in striatal mouse cells expressing mutant huntingtin, Nrf2 signalling is impaired, with decreased basal ARE reporter activity and impaired response to Nrf2 inducers [194,204], even though Nrf2 expression is unchanged [204]. This impaired Nrf2 signalling is analogous to similar impairments in amyotrophic lateral sclerosis.

In contrast to human tissue and genetic models of Huntington’s disease, Nrf2 signalling is generally increased in toxin models of the disease. Nrf2 signalling is induced in the penumbra surrounding malonate-induced striatal lesion, [205,206] and is accompanied by increased Nrf2 and HO1 expression but not NQO1 or GCLM [206]. In response to 3-nitropropionic acid, Nrf2 signalling is either increased or unchanged in the striatum of rats or mice, respectively [207,208,209]. In mouse and rat striatal slice cultures, quinolinic acid induces Nrf2 [210], while Nrf2 is increased by 3-nitropropionic acid in cultured brain cells [205,211]. The protective role of Nrf2 in toxin models of Huntington’s disease is further evidenced by studies utilising Nrf2^−/−^ mice, whereby deletion of Nrf2 renders mice more susceptible to 3-nitropropionic acid and malonate, with more severe motor impairment and increased striatal lesion size [205,211]. Nrf2-deficient primary neuronal cultures are also more susceptible to 3-nitropropionic acid toxicity [205]. These studies show that the Nrf2 pathway is functional and protective in toxin models of Huntington’s disease, in comparison to genetic models. This suggests that the impaired Nrf2 activity evident in human Huntington’s disease and genetic models is dependent on the presence of mutated huntingtin. The mechanism/s by which mutated huntingtin impairs Nrf2 signalling are unclear, but may involve the tumor suppressor protein homologous to the E6-AP carboxyl terminus domain and ankyrin repeat containing E3 ubiquitin-protein ligase 1 (HACE1), which has been found to facilitate Nrf2 signalling, and is decreased in the striatum of Huntington’s disease post-mortem brain and cultured mouse striatal cells expressing mutated huntingtin [194]. 

Although Nrf2 signalling is impaired in the presence of mutated huntingtin, pharmacological activators of Nrf2 are protective in animal models of Huntington’s disease (Table 6). Triterpenoids and dimethyl fumarate are protective in genetic models of Huntington’s disease, improving survival and motor function and increasing striatal neuron survival [212,213], and curcumin is effective in multiple models of Huntington’s disease [214,215,216]. Dimethyl fumarate, CDDO-methylamide, sulforaphane, curcumin nanoparticles, tert-butylhydroquinone, the citrus flavonone naringin, the ginseng extract protopanaxtriol, the organic disulphide cystamine and AI-3 are all protective against 3-nitropropionic acid and decrease striatal lesion size in rats and mice [25,70,207,208,209,211,217,218]. Deletion of Nrf2 abolishes the protective effects of cystamine and tert-butylhydroquinone, confirming the role of Nrf2 in these effects [25,211]. Thiazole-containing derivatives of 5-nitro-8-R-quinoline (e.g., MIND4) protect ex vivo rat brain slices transiently transfected with mutant huntingtin and a drosophila model of Huntington’s disease, and activate Nrf2 [219] in a mechanism involving Keap1 Cys-151 modification [84]. Finally, resveratrol is protective 3-nitropropionic acid [220], and is currently under investigation in a phase 3 clinical trial in Huntington’s disease patients (NCT02336633; Table 2).

### Cell Type-Specific Activation of Nrf2 in Huntington’s Disease

Nrf2 activation is not greatly altered in human Huntington’s disease and most genetic animal models of the disease. Therefore, it is not surprising that no evidence for the expression of Nrf2 in specific cell types has been reported for Huntington’s disease post-mortem tissue. While Nrf2 activation in oligodendrocytes or microglia from Huntington’s disease animal models has not been reported, Nrf2 signalling in mouse brain is induced in the penumbra surrounding malonate-induced striatal lesion, and this region colocalises with activated astrocytes [205,206]. In contrast, neurons but not astrocytes in the striatum of R6/2 mice are positive for Nrf2, and treatment dimethyl fumarate increases this neuronal Nrf2 [213]. 

Evidence for the protective role of Nrf2 activation in astrocytes is provided by overexpression studies. Astrocyte-specific overexpression of Nrf2 provides protection against malonate toxicity in mice, with decreased lesion size and greater Nrf2 activation [206], and adenoviral infection of astrocytes to overexpress Nrf2 in striatum reduces 3-nitropropionic acid lesion size [211]. Furthermore, transplantation of Nrf2-overexpressing astrocytes protects against malonate-induced damage in the striatum of mice, whereas wild-type astrocytes have no effect [205]. Similarly, Nrf2-overexpressing neural progenitor cells transplanted into striatum of wild-type mice differentiate into astrocytes and protect against malonate-induced damage [206]. Clearly, astrocytes are a valid therapeutic target for protective Nrf2 activation in the context of Huntington’s disease.

## 8. Multiple Sclerosis

Multiple sclerosis is a chronic inflammatory and demyelinating disease of the central nervous system characterised by focal lesions, demyelination of axons and inflammation involving infiltrating peripheral macrophages. As the lesions can occur at any location throughout the central nervous system, clinical symptoms are wide-ranging, commonly including autonomic, visual, motor and sensory disturbances. Multiple sclerosis has four clinical categories: relapsing-remitting, secondary progressive, primary progressive and progressive-relapsing. The former accounts for approximately 85% of cases, although patients can change between categories as symptoms change over time.

Nrf2 is consistently expressed in and around active lesions in post-mortem multiple sclerosis brain and spinal cord [221,222]. Elevated Keap1 is also evident at the edge of lesions [222]. The mRNA of Nrf2 target genes are upregulated in regions correlating with elevated Nrf2 protein, as is HO1 protein expression [222,223,224]. NQO1 and peroxiredoxin 2 are also strongly upregulated in lesions of post-mortem multiple sclerosis brain [225,226]. Clearly, Nrf2 activity is elevated in the vicinity of lesions in multiple sclerosis brain and spinal cord.

In contrast to human post-mortem analysis, reports of Nrf2 activity in animal models of multiple sclerosis are varied. In the cuprizone model of multiple sclerosis, Nrf2 and target proteins are decreased in the corpus callosum of mice treated for six weeks [227]. In contrast, Nrf2 and its targets are increased after 1–3 weeks of cuprizone treatment [228]. This likely reflects the progression of pathology and suggests that Nrf2 activation is an early event that can be overwhelmed or impaired as pathology progresses. In the experimental autoimmune encephalomyelitis model, Nrf2 and its targets are increased in the brains of mice and rats [229,230], whereas in the spinal cord, Nrf2 is most often decreased [231,232,233], but has also been reported as unchanged [234] or increased [235]. Deletion of Nrf2 worsens outcomes in the experimental autoimmune encephalomyelitis mouse model, with a more rapid and severe phenotype and greater microglial activation [236,237]. However, more recent reports detect robust worsening only in female mice [238], or no difference at all, at least at a single time-point [239]. Deletion of Nrf2 also exacerbates visual dysfunction in experimental autoimmune encephalomyelitis mice [238]. These discrepancies could represent regional differences in Nrf2 response to disease, but also likely reflect the inherent variability in multiple sclerosis models, in which clinical progression is impacted by species, strain and model conditions [240].

The best evidence for Nrf2 as a therapeutic target for the treatment of neurodegeneration comes from dimethyl fumarate. Dimethyl fumarate is clinically approved for the treatment of relapsing-remitting multiple sclerosis, following three successful phase 3 clinical trials [65], and is the only (widely accepted) Nrf2 inducer clinically approved for treatment of neurodegeneration (Table 2). Dimethyl fumarate activates Nrf2 and is protective in the experimental autoimmune encephalomyelitis model, diminishing symptom severity and preserving myelin and axon density and decreasing astrocyte activation, although macrophage infiltration is unaltered [237] (Table 7). These effects are abolished in Nrf2^−/−^ mice at a late stage (around 40d after initiation of the model) [237]. However, the presence of Nrf2 was recently found to have no impact on the protective effects of dimethyl fumarate in the acute phase (<21 d) of this model [239]. Furthermore, the anti-inflammatory effects of dimethyl fumarate in the acute phase are independent of the presence of Nrf2 [239]. Nevertheless, examination of blood from patients in the phase 3 clinical trials demonstrates results consistent with Nrf2 induction by dimethyl fumarate in the clinical population [241].

Natalizumab is an antibody against cell adhesion molecule α4-integrin that is clinically approved for the treatment of multiple sclerosis [98], and has been shown to activate Nrf2 and decrease markers of oxidative stress in peripheral blood mononuclear cells of multiple sclerosis patients [242], suggesting Nrf2 activation may be involved in its therapeutic action (Table 2). 

Other pharmacological inducers of Nrf2 have shown protective effects in the experimental autoimmune encephalomyelitis model of multiple sclerosis, including the triterpenoid CDDO-trifluoroethylamide (also effective in the lysophosphatidyl choline model) [234], 3H-1,2-dithiole-3-thione [243], sulforaphane [230], the natural alkaloid matrine [244] and TFM-735, which was identified from a chemical library screen [245] (Table 7). Resveratrol [246,247,248,249,250,251,252] and curcumin [253,254] are effective in several models of multiple sclerosis, although the role of Nrf2 was not considered in these studies. Nevertheless, these compounds are known to activate Nrf2, among other actions [142,143]. Polymerized nanocurcumin, designed to increase the bioavailability of curcumin, is effective in the rat experimental autoimmune encephalomyelitis model [235]. Curcumin plus interferonβ-1a, and polymerized nanocurcumin are currently being investigated in phase 2 clinical trials for the treatment of multiple sclerosis (NCT01514370, NCT03150966; Table 2). 

Other treatments that are effective in the experimental autoimmune encephalomyelitis model have been shown to activate or restore Nrf2 activity, including physical exercise [232] and peripheral administration of human mesenchymal stem cells [231]. The natural flavonoid myricetin activates Nrf2 and alleviates cuprizone-induced motor and behavioural impairments in mice [227].

### Cell Type-Specific Activation of Nrf2 in Multiple Sclerosis

In contrast to other diseases, cell-specific activation of Nrf2 has been well-studied in post-mortem multiple sclerosis tissue. Elevated expression of Nrf2 is found in astrocytes and infiltrating macrophages in active lesions [221], and in oligodendrocytes at lesion edges [222]. Elevated Nrf2 is only occasionally expressed in neurons, even when surrounded by Nrf2-positive glia [222], and is found in some spinal cord neurons [237]. Keap1 expression is elevated in astrocytes, microglia and oligodendrocytes at lesion edges [222]. 

Upregulation of Nrf2 targets tends to mirror Nrf2 activation as expected. NQO1 is strongly upregulated in lesions of post-mortem multiple sclerosis brain, predominantly in astrocytes and some macrophages and oligodendrocytes, whereas elevated NQO1 in neurons is rare [225,226]. HO1 protein is elevated in astrocytes, oligodendrocytes and microglia at lesions [222,223,229], and peroxiredoxin 2 is strongly upregulated in lesions predominantly in astrocytes, although also upregulated in neurons in grey matter lesions [226]. 

Evidence for cell-specific Nrf2 activation in animal models of multiple sclerosis is more scarce than in post-mortem human tissue, perhaps reflecting the variability of Nrf2 activity in these studies. In the experimental autoimmune encephalomyelitis model, Nrf2 is present in spinal cord motor neurons, but not glia [237]. However, HO1 protein is elevated in astrocytes and microglia/macrophages in lesions in this model [229]. In the cuprizone model, most Nrf2-positive cells are identified as oligodendrocytes, and occasionally astrocytes [228].

Overall, the evidence for cell-specific activation of Nrf2 in multiple sclerosis indicates a clear tendency for glial activation. Of note, this is the only disease discussed in this review in which robust Nrf2 activation is observed in oligodendrocytes or microglia. This may reflect the demyelinating and strongly inflammatory nature, respectively, of multiple sclerosis. However, it clearly demonstrates that these cells are capable of increasing Nrf2 activity in response to disease.

Astrocyte-specific activation of Nrf2 via targeted ablation of Keap1 protects against the cuprizone model of multiple sclerosis [228] (Table 7). This treatment prevents oligodendrocyte loss and axonal damage, and decreases microgliosis [228], demonstrating that astrocyte-specific Nrf2 activation is sufficient to prevent pathology in the cuprizone model. Cell type-specific activation of Nrf2 has not been assessed for pharmacological inducers of Nrf2 in multiple sclerosis models, with the exception of dimethyl fumarate. In experimental autoimmune encephalomyelitis model mice, dimethyl fumarate appears to elevate Nrf2 in neurons, astrocytes and oligodendrocytes, but not microglia/macrophages [237]. Hence it is not clear which cells are targeted by most pharmacological Nrf2 inducers in models of multiple sclerosis, but may include astrocytes, neurons and oligodendrocytes.

## 9. Reconciling In Vitro and In Vivo Evidence for Neuronal Nrf2 Expression

Robust Nrf2 activation in response to inducers is often observed in primary neuronal cultures [205,255,256,257]. However, the cultures in these studies always contain at least a minor population of astrocytes. When specific markers are used, it is clear that strong Nrf2 activation is restricted to the astrocytes in these cultures [25,147,205,256,258]. Detailed analysis of pure neuronal, astrocyte and mixed cultures clearly demonstrates that Nrf2 activation is not observed in pure primary neuron cultures that lack astrocytes, and the extent of Nrf2 activation is dependent on the abundance of astrocytes [259]. Pharmacological activation of Nrf2 protects mixed cultures from oxidative stress; however, inhibition of Nrf2 specifically in astrocytes in these cultures abolishes the protective effect of the Nrf2 inducer, indicating that Nrf2 activation solely in astrocytes is sufficient and necessary to protect neurons from oxidative stress [258]. Interestingly, while treatment of mixed cultures with an Nrf2 inducer upregulates Nrf2 target genes predominantly in astrocytes, some Nrf2 target genes are also upregulated in neurons. These neuronal gene changes are abolished when Nrf2 is inhibited specifically in the co-cultured astrocytes, indicating that the neuronal changes are dependent on altered astrocytic Nrf2 activity and not a direct action of the Nrf2 inducer in neurons [258]. This indicates that Nrf2 activation in astrocytes can influence neuronal antioxidant gene expression. 

In contrast to primary neurons, Nrf2 activation is often observed in neuronal cell lines (e.g., [13,69]). However, cell lines differ from bona fide neurons in many respects, not least their ability to proliferate as compared to neurons which are post mitotic. Indeed, Nrf2 expression appears to be specifically repressed in neurons in a mechanism facilitating neuronal development [259]. This is evident in cultured mouse and human neurons, and in neurons isolated from young adult mouse brain [259]. Hence, Nrf2 activity in neuronal cell lines may not accurately reflect the Nrf2 activity of neurons in vivo.

Although Nrf2 is more often expressed in astrocytes, neuronal Nrf2 activation is apparent in humans and mice, in neurodegeneration and normal aging. This appears to contradict the findings that Nrf2 expression is repressed in cultured neurons. This can be partially explained by findings that synaptic activity can upregulate Nrf2 target genes in neurons cultured in the absence of astrocytes and when Nrf2 is deleted [260], and specific astrocytic Nrf2 activation can upregulate Nrf2 target genes in neurons [258], indicating that at least in culture, some Nrf2 target genes can be regulated in neurons independently of direct neuronal Nrf2 activation. While this can potentially explain the in vivo alterations in Nrf2 target genes in neurons, it cannot explain the accumulation of Nrf2 protein. A possible explanation for this is that many of the results indicating lack of neuronal Nrf2 activation are generated in cells cultured from embryonic or neonatal mice, or at best young adult mouse brain [259]. However, Nrf2 expression is observed in neurons of aged human and mouse brain. Perhaps neurons only express Nrf2 in later adulthood. Nevertheless, even though Nrf2 protein can be detected in neurons in aged human and mouse brain, this is rarely accompanied by increased Nrf2 target expression, with the exception of Alzheimer’s disease. Hence, it appears that the elevated Nrf2 protein rarely results in functional Nrf2 signalling and most evidence suggests that Nrf2 signalling is only robustly activated in glia, and astrocytes in particular.

That Nrf2 inducers only activate Nrf2 in astrocytes and not neurons in vitro suggests that the protective effects of Nrf2 inducers in animal models of neurodegeneration are due to Nrf2 activation specifically in astrocytes. Although rarely specifically investigated, the available evidence is mixed. The increased expression of HO1 in mice treated with sulforaphane or 2′,3′-dihydroxy-4′,6′-dimethoxychalcone in Parkinson’s disease models is restricted to astrocytes and microglia, and not expressed in neurons [60,85]. The Nrf2 inducer pyrrolidine dithiocarbamate, protective in an Alzheimer’s disease animal model [142], activates Nrf2 in astrocytes but not neurons in vitro and in vivo [147]. In contrast, dimethyl fumarate activates Nrf2 in neurons in mouse models of Parkinson’s disease and Huntington’s disease [66,213], and neurons, astrocytes and oligodendrocytes but not microglia in models of multiple sclerosis [237]. Together, this limited evidence suggests that Nrf2 inducers differentially target cell populations, and that the cell types targeted are dependent on disease conditions. Therefore, it remains unclear which cell type is the predominant target of small molecule Nrf2 inducers. 

Overexpression studies do not provide insight into which cells endogenously express Nrf2, but they do provide proof-of-concept data for which cells are capable of preventing neuronal dysfunction. Overexpression of Nrf2 in neurons is protective in mice modelling Alzheimer’s disease [118], but not amyotrophic lateral sclerosis [161]. This suggests that there are regional and disease-specific differences in the response of neurons to neuronal Nrf2 activation. In contrast, overexpression of Nrf2 in astrocytes is protective in models of Parkinson’s disease [20,24], amyotrophic lateral sclerosis [18], Huntington’s disease [206,211], and multiple sclerosis [228], indicating that activation of Nrf2 in astrocytes is a viable therapeutic target for these diseases. 

## 10. Cautionary Note on Nrf2 Activation

This review describes Nrf2 activity in the context of neurodegeneration, and the therapeutic potential of targeting its activation. However, over-activation or prolonged activation of Nrf2 can be detrimental. Prolonged activation of Nrf2 in drosophila shortens lifespan [261] and promotes the malignant transformation of human cells [262]. Similarly, mutations in Nrf2 that disrupt its recognition by Keap1 render it constitutively active and promote cancer survival in humans [263]. Indeed, Nrf2 is highly expressed by many cancers, which contributes to their resistance to anti-cancer treatments [264]. In addition, many Nrf2 inducers are electrophiles and activate Nrf2 by modifying Keap1 cysteine residues to permit Nrf2 dissociation and stabilisation. However, these inducers can also react with other molecules. Highly potent Nrf2 activation by triterpenoid-based compounds are very effective in preclinical models of neurodegeneration [69,119,168], but the clinical derivative bardoxolone methyl has toxicity issues in humans due to off-target effects [265]. Targeting other aspects of Nrf2 regulation such as Bach1 [13] may be more effective. Hence, therapeutic agents targeting Nrf2 must have high specificity for Nrf2 activation, and their use for the treatment of neurodegeneration must be considered against the potential negative consequences of Nrf2 activation such as cancer promotion.

## 11. Conclusions

Each neurodegenerative disease discussed in this review has a particular Nrf2 phenotype. It appears that Nrf2 is activated in astrocytes in Parkinson’s disease, in astrocytes, neurons and possibly microglia in Alzheimer’s disease, and in all glial cells but not neurons in multiple sclerosis. Amyotrophic lateral sclerosis and Huntington’s disease appear to exhibit impaired Nrf2 signalling, while the evidence for Nrf2 activation in aging is minimal and unclear. Overall, while Nrf2 expression is evident in neurons in aging and neurodegeneration, it is often more strongly activated in astrocytes. Neurodegeneration is most often accompanied by elevated Nrf2 activity in regions of most severe pathology. Generally, deletion of Nrf2 usually worsens disease phenotype, whereas overexpression or pharmacological activation of Nrf2 is protective in animal models of neurodegeneration, although there are important exceptions. These results clearly indicate that the endogenous Nrf2 response to disease is insufficient to prevent oxidative stress and neuronal damage, and that Nrf2 is a valid therapeutic target for the treatment of neurodegeneration. 

It is unclear why endogenous Nrf2 activation is insufficient in neurodegeneration, but at least for amyotrophic lateral sclerosis and Huntington’s disease, the available evidence suggests endogenous Nrf2 signalling is impaired in these diseases at the molecular level, involving aberrant TDP-43 or huntingtin, respectively. Whether analogous impairments exist for other neurodegenerative diseases is yet to be established, although transcriptomic changes in Nrf2^−/−^ mice closely mirror those in human Alzheimer’s disease [15], suggesting that Nrf2 signalling is also impaired in Alzheimer’s disease. Nevertheless, pharmacological activation of Nrf2 is almost unfailingly protective in every model of neurodegeneration assessed, as is overexpression of Nrf2 specifically in astrocytes, clearly indicating the therapeutic potential of Nrf2 activation for the treatment of neurodegeneration. Furthermore, several drugs that can activate Nrf2 are clinically approved and several more are currently undergoing clinical trials.

Nevertheless, many questions remain regarding Nrf2 in neurodegeneration. The requirement of Nrf2 for the protective effect of pharmacological Nrf2 inducers is only rarely assessed, and has only been assessed in acute models of Parkinson’s disease, Huntington’s disease and multiple sclerosis. This requirement of Nrf2 is yet to be assessed in any models of Alzheimer’s disease or amyotrophic lateral sclerosis. The latter likely reflects the inconvenience of breeding transgenic disease-model mice lacking Nrf2, but such mice have been reported for both Alzheimer’s disease [15] and amyotrophic lateral sclerosis [159,161,162] models. Nrf2 activation is currently only poorly characterised in Huntington’s disease, and its activity remains to be determined in human Huntington’s disease brain tissue. There is also only minimal evidence suggesting Nrf2 is impaired. Assessing the influence of Nrf2 deletion in genetic models of Huntington’s disease would provide further insight into a potential mutant huntingtin-dependent mechanism of Nrf2 impairment, reminiscent of amyotrophic lateral sclerosis, in which Nrf2 deletion has little impact on the survival of disease model mice. As for amyotrophic lateral sclerosis, Nrf2 induction should be assessed in non-SOD1 models. That this has not been reported yet is likely due to the poor performance of alternate models to date [266], although better models are being developed [267,268], which should be amenable to preclinical drug trials. In terms of elucidating which cells are most important for Nrf2 activation, it would be pertinent to examine Nrf2 overexpression in neurons in diseases other than Alzheimer’s disease, and to assess Nrf2 overexpression in microglia or oligodendrocytes. Lastly, the response of Nrf2 to normal aging requires further analysis. Longitudinal studies investigating Nrf2 activation in various regions would address this.

Although Nrf2 expression is repressed in cultured neurons, the evidence indicates that endogenous Nrf2 is activated in neurons in neurodegeneration and aging. In contrast, Nrf2 is readily inducible in cultured astrocytes, and tends to be more strongly activated in neurodegeneration in vivo. That Nrf2 is more readily activated in cultured astrocytes suggests that most pharmacological Nrf2 inducers may be specifically targeting astrocytes in vivo, although there is very limited direct evidence for this. Furthermore, with the exception of multiple sclerosis, there is little data on Nrf2 activation in microglia and particularly oligodendrocytes in human tissue or animal models of neurodegeneration. Given that microglia clearly exhibit functional Nrf2 signalling [269], and the universal presence of glial activation in these diseases [270], microglia may be an important target for Nrf2 induction in neurodegeneration. Similarly, endogenous Nrf2 activation is clearly evident in oligodendrocytes in multiple sclerosis [222], yet Nrf2 signalling in oligodendrocytes has not been assessed in any other disease. This may be pertinent for amyotrophic lateral sclerosis, which also involves demyelination [271]. Further evidence is required to determine in which cells Nrf2 activation occurs in response to treatments targeting Nrf2. This will provide insight into the molecular mechanisms driving disease and opportunities for the treatment of neurodegeneration.

## Figures and Tables

**Table 1:** Preclinical studies on Nrf2 activation in animal models of Parkinson’s disease.

Treatment	Model	Species	Nrf2^−/−^ Assessed?	References
Dimethyl fumarate	MPTP	Mouse	Yes	[13]
	MPTP	Mouse	No	[66]
	6OHDA	Mouse	No	[68]
	α-synuclein	Mouse	Yes	[67]
CDDO–MA	MPTP	Mouse	No	[70]
CDDO–EA	MPTP	Mouse	Yes	[69]
CDDO–TFEA	MPTP	Mouse	Yes	[69]
3H-1,2-dithiole-3-thione	MPTP	Mouse	Yes	[72]
Sulforaphane	MPTP	Mouse	Yes	[60]
	6OHDA	Mouse	No	[61]
	Rotenone	Mouse	No	[62]
	α-synuclein	Drosophila	No	[63]
Curcumin	Rotenone	Rat	Nrf2-kd	[56]
	Rotenone	Mouse	No	[71] *
Resveratrol	Rotenone	Rat	No	[74]
	MPTP	Mouse	No	[75] *
	MPTP	Mouse	No	[76] *
	6OHDA	Rat	No	[77] *
Carnosic acid	6OHDA	Rat	No	[73]
MIND4	MPTP	Mouse	No	[84]
2′,3′-dihydroxy-4′,6′-dimethoxychalcone	6OHDA	Mouse	No	[85]
Cu^II^(atsm)	MPTP	Mouse	No	[78] *
	6OHDA	Mouse	No	[78] *
	α-synuclein	Mouse	No	[78] *
	α-synuclein + MPTP	Mouse	No	[78] *
Keap1-kd	MPTP	Mouse	No	[83]
Transplanted Nrf2-overexpressing astrocytes	6OHDA	Mouse	n.a.	[57]
Nrf2 overexpression in astrocytes	MPTP	Mouse	n.a.	[24]
Nrf2 overexpression in astrocytes in Nrf2^−/−^ mice	MPTP	Mouse	n.a.	[24]
Nrf2 overexpression in astrocytes	α-synuclein	Mouse	n.a.	[20]

* Not assessed in the context of Nrf2; n.a.: Not applicable.

**Table 2:** Clinical trials involving Nrf2 inducers for neurodegenerative diseases.

Drug	NCT ^#^	Study Name	Phase	# of Patients	Efficacy?	References
*Parkinson’s disease*						
Cu^II^(atsm) *	03204929	n.p.	1	38 ^c^	Ongoing	
*Alzheimer’s disease*						
Curcumin	00099710	n.p.	2	36	No	[86]
	n.p.	n.p.	2	34	No	[87]
dl-3-n-butylphthalide	02711683	n.p.	Obs	120 ^c^	Ongoing	
Resveratrol	01504854	n.p.	2	119	Yes ^#^	[88,89]
*Amyotrophic lateral sclerosis*						
Edaravone ^a,^*	01492686	MCI186-19	3	137	Yes	[90]
	00330681	MCI186-16	3	206	No	[91]
	00415519	MCI186-18	3	25	No	[92]
Cu^II^(atsm) *	02870634	n.p.	1	50 ^c^	Ongoing	
	03136809	n.p.	1/2	50 ^c^	Ongoing	
*Huntington’s disease*						
Resveratrol	02336633	REVHD	3	102 ^c^	Ongoing	
*Multiple sclerosis*						
Dimethyl fumarate ^b^	00420212	DEFINE	3	1234	Yes	[93]
	00451451	CONFIRM	3	1417	Yes	[94]
	00835770	ENDORSE	3	229	Yes	[95]
Natalizumab ^b,^*	00027300	AFFIRM	3	942	Yes	[96]
	00030966	SENTINEL	3	1171	Yes	[97]
	01416181	ASCEND	3	887	No	[98]
Nanocurcumin	03150966	n.p.	2	50 ^c^	Ongoing	
Curcumin + IFNbeta 1a	01514370	CONTAIN	2	80	Ongoing	

^a^ Clinically approved for treatment of amyotrophic lateral sclerosis; ^b^ Clinically approved for treatment of relapsing-remitting multiple sclerosis; ^c^ Estimated enrolment; ^#^ Efficacy detected in a subsequent analysis of a subset of patients [89]; * Nrf2 activation has been reported (see text) but not recognised as a primary action; Obs: Observational study; n.p.: None provided.

**Table 3:** Alterations in Nrf2 and Nrf2 targets in post-mortem Alzheimer’s disease brain regions.

Nrf2 Target	Alteration in Alzheimer’s Disease	Method of Detection	References
*Hippocampus*			
Nrf2	Increased	IHC, WB	[9]
	Increased	WB	[100]
	Decreased	IHC	[36]
HO1	Increased	WB	[9]
	Increased	IHC	[101]
	Increased	IHC, WB	[102]
	Increased	IHC	[103]
	Increased	IHC, WB	[100]
NQO1	Increased	IHC	[104]
	Increased	IHC, WB, activity	[105]
p62	Increased	WB	[9]
Glutathione reductase	Increased	mRNA	[106]
Glutathione peroxidase	Increased	mRNA	[106]
Glutathione-*S*-transferase	Unchanged	mRNA	[106]
	Decreased	WB, activity	[107]
Thioredoxin	Decreased	DB	[108]
Thioredoxin reductase	Unchanged	Activity	[108]
*Temporal cortex*			
Keap1	Unchanged	IHC	[37]
	Mislocalised	WB	[37]
HO1	Increased	mRNA	[37]
	Increased	IHC	[101]
	Increased	IHC, WB	[102]
NQO1	Increased (ns)	mRNA	[37]
GCLM	Increased	mRNA	[37]
p62	Increased	mRNA	[37]
Glutathione-*S*-transferase	Decreased	WB, activity	[107]
Thioredoxin	Decreased	DB	[108]
Thioredoxin reductase	Unchanged	Activity	[108]
*Frontal cortex*			
Nrf2	Decreased	WB	[36]
NQO1	Increased	IHC, activity	[109]
GCLC	Decreased	WB	[110]
Glutathione synthetase	Decreased	WB	[110]
Glutathione reductase	Increased	WB	[110]
Glutathione-*S*-transferase	Unchanged	2D-E	[111]
Peroxiredoxin 1	Unchanged	2D-E	[111]
Peroxiredoxin 2	Increased	2D-E	[111]
Peroxiredoxin 3	Unchanged	2D-E	[111]
Peroxiredoxin 6	Unchanged	2D-E	[111]

2D-E, two-dimensional electrophoresis followed by mass spectrometry; DB, dot blot; IHC, immunohistochemistry; ns, not significant; WB, western blot.

**Table 4:** Preclinical studies on Nrf2 activation in animal models of Alzheimer’s disease.

Treatment	Model	Species	Improved Cognition?	References
CDDO–MA	Tg19959	Mouse	Yes	[119]
dl-3-n-butylphthalide	APP/PS1	Mouse	Yes	[120]
Sulforaphane	Amyloid-β	Mouse	Yes	[123]
	d-galactose and aluminium	Mouse	Yes	[127] *
Curcumin ^a^	Tg2576	Mouse	n.d.	[128] *
	Amyloid-β	Rat	Yes	[129] *
	Tg2576	Mouse	n.d.	[130] *
	APP/PS1	Mouse	n.d.	[131] *
	Amyloid-β	Rat	Yes	[132] *
	APP/PS1	Mouse	Yes	[133] *
	5xFAD	Mouse	Yes	[134] *
	5xFAD	Mouse	Yes	[135] *
Resveratrol	Tg19959	Mouse	n.d.	[136] *
	p25	Mouse	Yes	[137] *
	Amyloid-β	Rat	Yes	[138]
	SAMP8	Mouse	Yes	[139] *
	APP/PS1	Mouse	Yes	[140] *
	Amyloid-β	Mouse	Yes	[141] *
Methysticin	APP/PS1	Mouse	Yes	[121]
Sodium arsenite	Amyloid-β	Rat	Yes	[114]
Carnosic acid	hAPP-J20	Mouse	Yes	[122]
	3xTg	Mouse	n.d.	[122]
Pyrrolidine dithiocarbamate	APP/PS1	Mice	Yes	[142] *
Keap1 deletion	Amyloid-β expression	Drosophila	Increased lifespan and motor function	[124]
Lentiviral transduction of Nrf2 in hippocampal neurons	APP/PS1	Mouse	Yes	[118]

^a^ Selected studies, see [143] for further examples; * Not assessed in the context of Nrf2; FAD: familial Alzheimer’s disease; n.d.: Not determined; SAMP8: senescence accelerated mouse prone 8.

**Table 5:** Preclinical studies on Nrf2 activation in animal models of amyotrophic lateral sclerosis.

Treatment	Model	Species	Improved Motor Function?	Extends Survival?	References
CDDO–EA	SOD1-G93A	Mouse	Yes	Yes	[168]
CDDO–TFEA	SOD1-G93A	Mouse	Yes	Yes	[168]
dl-3-n-butylphthalide	SOD1-G93A	Mouse	Yes	Yes	[169]
Resveratrol	SOD1-G93A	Mouse	No	No	[170] *
	SOD1-G93A	Mouse	Yes	Yes	[171] *
	SOD1-G93A	Mouse	Yes	Yes	[172] *
	SOD1-G93A	Mouse	Yes	Yes	[173] *
CPN-9	SOD1-H46R	Mouse	Yes	Yes	[174]
WN1316	SOD1-G93A	Mouse	Yes	Yes	[175]
	SOD1-H46R	Mouse	Yes	Yes	[175]
S[+]-apomorphine	SOD1-G93A	Mouse	Yes	No	[176]
Cu^II^(atsm)	SOD1-G93A	Mouse	Yes	Yes	[180] *
	SOD1-G37R	Mouse	Yes	Yes	[181] *
	SOD1-G37R	Mouse	Yes	n.d.	[182] *
	SOD1-G93A	Mouse	Yes	Yes	[183] *
	SOD1-G93AxCCS	Mouse	n.d.	Yes	[183] *
	SOD1-G93A	Mouse	Yes	Yes	[184] *
	SOD1-G93A	Mouse	Yes	Yes ^#^	[185] *
Pyrrolidine dithiocarbamate	SOD1-G93A	Rat	No	No	[177] *
Lentiviral delivery of Nrf2/EAAT2/GDH2	SOD1-G93A	Mouse	Yes	Yes	[179]
Nrf2 overexpression in astrocytes	SOD1-G93A	Mouse	Yes	Yes	[18]
	SOD1-H46R/H48Q	Mouse	n.d.	Yes	[18]
AAV6 intramuscular delivery of Nrf2	SOD1-G93A	Mouse	No	No	[186]
Nrf2 overexpression in skeletal muscle	SOD1-G93A	Mouse	n.d.	No	[161]
Nrf2 overexpression in neurons	SOD1-G93A	Mouse	n.d.	No	[161]

^#^ 5% increase in survival, not significant; * Not assessed in the context of Nrf2; n.d.: Not determined.

**Table 6:** Preclinical studies on Nrf2 activation in animal models of Huntington’s disease.

Treatment	Model	Species	Nrf2^−/−^ Assessed?	References
Dimethyl fumarate	R6/2	Mouse	No	[213]
	YAC128	Mouse	No	[213]
	3-NP	Mouse	No	[209]
CDDO–MA	3-NP	Rat	No	[70]
CDDO–EA	N171-82Q	Mouse	No	[212]
CDDO–TFEA	N171-82Q	Mouse	No	[212]
Sulforaphane	3-NP	Mouse	No	[209]
Curcumin	CAG140	Mouse	No	[214] *
	mHTT exon1	Drosophila	No	[215] *
	Quinolinic acid	Rat	No	[216] *
Curcumin nanoparticles	3-NP	Rat	No	[217]
Resveratrol	3-NP	Mouse	No	[220] *
tert-Butylhydroquinone	3-NP	Mouse	Yes	[211]
	3-NP	Rat	No	[218]
Naringin	3-NP	Rat	No	[207]
Protopanaxtriol	3-NP	Rat	No	[208]
AI-3	3-NP	Mouse	No	[209]
Cystamine	R6/2	Mouse	No	[198] *
	3-NP	Mouse	Yes	[25]
MIND4	mHTT exon1	Rat brain slice	No	[219]
	mHTT exon1	Drosophila	No	[219]
Adenoviral delivery of Nrf2 to astrocytes	3-NP	Mouse	n.a.	[211]
Transplanted Nrf2-overexpressing astrocytes	Malonate	Mouse	n.a.	[205]
Transplanted Nrf2-overexpressing neural progenitor cells	Malonate	Mouse	n.a.	[206]
Overexpression of Nrf2 in astrocytes	Malonate	Mouse	n.a.	[206]

3-NP: 3-Nitropropionic acid; * Not assessed in the context of Nrf2; n.a.: Not applicable.

**Table 7:** Preclinical studies on Nrf2 activation in animal models of multiple sclerosis.

Treatment	Model	Species	Nrf2^−/−^ Assessed?	References
Dimethyl fumarate ^a^	EAE	Mouse	Yes	[237]
	EAE	Mouse	Yes	[239]
CDDO–TFEA	EAE	Mouse	No	[234]
	LPC	Rat	No	[234]
3H-1,2-dithiole-3-thione	EAE	Mouse	No	[243]
Sulforaphane	EAE	Mouse	No	[230]
Curcumin	EAE	Mouse	No	[253] *
	EAE	Mouse	No	[254] *
Nanocurcumin	EAE	Rat	No	[235]
Resveratrol	EAE	Mouse	No	[246] *
	EAE	Mouse	No	[247] *
	EAE	Mouse	No	[248] *
	EAE	Mouse	No	[249] *
	EAE ^#^	Mouse	No	[250] *
	TMEV-IDD ^#^	Mouse	No	[250] *
	Cuprizone	Mouse	No	[251] *
	EAE	Mouse	No	[252] *
Matrine	EAE	Rat	No	[244]
TFM-735	EAE	Mouse	No	[245]
Myricetin	Cuprizone	Mouse	No	[227]
Physical exercise	EAE	Mouse	No	[232]
Mesenchymal stem cells	EAE	Mouse	No	[231]
Astrocyte-specific Keap1-ko	Cuprizone	Mouse	No	[228]

^a^ Clinically approved for the treatment of relasping-remitting multiple sclerosis; ^#^ Worsened clinical outcome; * Not assessed in the context of Nrf2; EAE: experimental autoimmune encephalomyelitis; LPC: lysophosphatidyl choline; TMEV-IDD: Theiler’s murine encephalomyelitis virus-induced demyelinating disease.
